# Infrared Thermography in Symptomatic Knee Osteoarthritis: Joint Temperature Differs Based on Patient and Pain Characteristics

**DOI:** 10.3390/jcm12062319

**Published:** 2023-03-16

**Authors:** Luca De Marziani, Angelo Boffa, Lucia Angelelli, Luca Andriolo, Alessandro Di Martino, Stefano Zaffagnini, Giuseppe Filardo

**Affiliations:** 1 Clinica Ortopedica e Traumatologica 2, IRCCS Istituto Ortopedico Rizzoli, 40136 Bologna, Italy; 2 Applied and Translational Research (ATR) Center, IRCCS Istituto Ortopedico Rizzoli, 40136 Bologna, Italy

**Keywords:** infrared thermography, knee, osteoarthritis, joint temperature, inflammation, neuropathic pain

## Abstract

The aim of this study was to evaluate osteoarthritis (OA) patients with infrared thermography to investigate imaging patterns as well as demographic and clinical characteristics that influence knee inflammation. Forty patients with one-sided symptomatic knee OA were included and evaluated through knee-specific PROMs and the PainDETECT Questionnaire for neuropathic pain evaluation. Thermograms were captured using a thermographic camera FLIR-T1020 and temperatures were extracted using the software ResearchIR for the overall knee and the five ROIs: medial, lateral, medial patella, lateral patella, and suprapatellar. The mean temperature of the total knee was 31.9 ± 1.6 °C. It negatively correlated with age (rho = −0.380, *p* = 0.016) and positively correlated with BMI (rho = 0.421, *p* = 0.007) and the IKDC objective score (tau = 0.294, *p* = 0.016). Men had higher temperatures in the knee medial, lateral, and suprapatellar areas (*p* = 0.017, *p* = 0.019, *p* = 0.025, respectively). Patients with neuropathic pain had a lower temperature of the medial knee area (31.5 ± 1.0 vs. 32.3 ± 1.1, *p* = 0.042), with the total knee negatively correlating with PainDETECT (*p* = 0.045). This study demonstrated that the skin temperature of OA symptomatic knees is influenced by demographic and clinical characteristics of patients, with higher joint temperatures in younger male patients with higher BMI and worst objective knee scores and lower temperatures in patients affected by neuropathic pain.

## 1. Introduction

Osteoarthritis (OA) is a common form of degenerative joint disease affecting the adult population, characterized by articular cartilage loss within the synovial joints and associated with hypertrophy of the bone (osteophytes and subchondral bone sclerosis), thickening of the capsule, and synovial inflammation [1]. An important aspect of OA is the interpatient variability in clinical and structural manifestations [2,3]. This heterogeneity may be one of the major factors associated with the complexity of OA management and the difficulties in developing one-fits-all therapeutic strategies. In fact, nowadays, no conservative therapies have been proven to arrest or modify the disease progression, nor to be highly effective for symptomatic relief [4,5]. For this reason, the identification of specific OA features could help to better identify different diseases patterns and target treatments and manage each patient according to the specific disease phase and manifestation [6]. Among the key aspects investigated, lot of attention has been placed on the inflammatory process involving OA joints.

Inflammation plays a central role in the pathophysiology of OA, with the involvement of the synovial membrane and the release of pro-inflammatory cytokines. These factors induce chondrocytes to produce degradative enzymes of the extracellular matrix and inhibit both tissue repair and regeneration [7]. During the inflammatory process, there is an increase of blood flow that can manifest clinically with redness and heat, as well as with joint swelling and pain [8]. As many available treatments target the inflammatory process, the quantification of the inflammatory component in the OA process could help to better characterize patients with OA, favoring a more targeted treatment [9,10]. In this light, the temperature is a key physical property, as its values detected by infrared thermography could reflect the articular inflammatory process [11]. Therefore, infrared thermography has been proposed as a tool for OA diagnosis and monitoring of disease state, progression, and response to medical treatment, in particular in relation to the inflammatory components [12]. However, although the use of infrared thermography for the evaluation of patients with OA is growing, data are still sparse and evidence on thermographic findings in OA patients is lacking.

The aim of this study was to evaluate OA patients with infrared thermography, to investigate imaging patterns as well as demographic and clinical characteristics that could influence the skin temperature of the knee of patients affected by symptomatic OA.

## 2. Materials and Methods

This study was approved by the hospital ethics committee of the IRCCS Istituto Ortopedico Rizzoli, Italy (n. 0017413). Patients were enrolled by orthopedic physicians between December 2021 and April 2022 in a research outpatient clinic focused on patients with knee OA. Informed consent was obtained from each patient for study participation. Male or female patients with one-sided symptomatic knee OA (Kellgren–Lawrence grade ≥ 2) with a history of chronic pain or swelling (at least 6 months) were included in the study. The following exclusion criteria were used for selection: history of trauma or intra-articular injection therapy within 6 months before treatment or knee surgery within 12 months; presence of any concomitant knee lesion causing pain or swelling, neoplasms, dermatological and vascular conditions, systemic disorders (e.g., uncontrolled diabetes), metabolic disorders of the thyroid, severe cardiovascular diseases, rheumatoid arthritis, inflammatory arthropathy, hematological diseases, infections, immunodepression, antidepressant, anticoagulants, or antiaggregant therapy; and use of nonsteroidal anti-inflammatory drugs in the 5 days before the investigation.

Forty consecutive patients were enrolled according to the inclusion/exclusion criteria. Among them, 26 patients were men and 14 women, with a mean age of 61.3 ± 9.3 years and a mean body mass index (BMI) of 25.2 ± 3.0. All demographic and clinical patients’ characteristics are reported in Table 1.

After enrollment in the study and just before the infrared thermography evaluation, patients were clinically assessed thorough knee-specific patient reported outcome measurements (PROMs) including the International Knee Documentation Committee (IKDC) subjective and objective scores, the Knee injury and Osteoarthritis Outcome Score (KOOS) sub-scales, the EuroQol Visual Analog Scale (EQ-VAS), the Tegner score, the Visual Analogue Scale (VAS) for the symptomatic knee pain, and the PainDETECT Questionnaire for the neuropathic pain evaluation. For the last score, patients with values lower than 13 were considered negative for neuropathic pain, while patients with values higher or equal to 13 were considered positive for neuropathic pain. Subjective clinical questionnaires were compiled by patients with the support of the clinician, while the IKDC objective score was evaluated by the clinician. Moreover, all participants underwent weight-bearing antero-posterior to assess the baseline OA severity according to the Kellgren–Lawrence classification. Finally, the skin temperature of the knee affected by symptomatic OA was evaluated with thermography imaging as reported below. After data collection, further analyses were performed to determine the demographic and clinical parameters that influenced the skin temperature of the OA knee.

### 2.1. Infrared Imaging Procedure and Analysis

**Figure 1:**
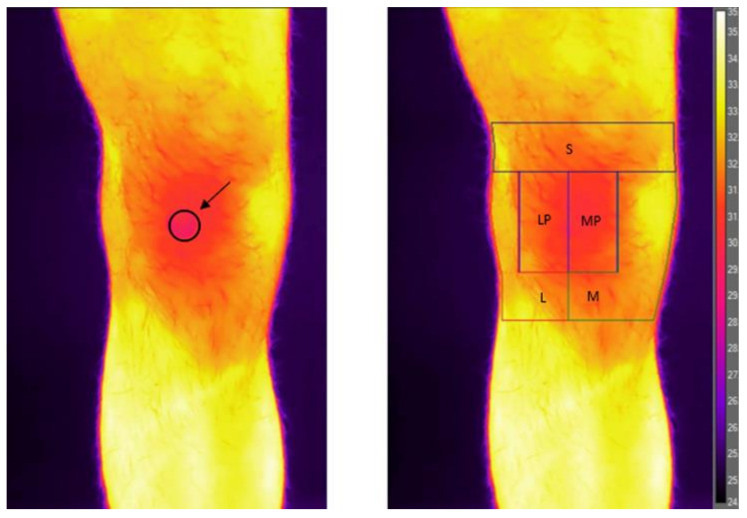
Infrared thermography analysis. On the left, the infrared image obtained with the patellar marker (identified with circle and arrow), while on the right, the image obtained with the considered region of interest (ROI): L, lateral; LP, lateral patella; M, medial; MP, medial patella; and S, suprapatellar.

The infrared imaging evaluation was performed in a dedicated outpatient clinic shielded from direct sunlight and with a temperature controlled and set at 23.0 °C and a mean humidity of 45 ± 3%. The image acquisition was always performed in the same time slot between 14:00 pm and 17:00 pm in order to minimize the circadian variations of the temperature. According to Marins et al. [13], participants were asked to sit for ten minutes without touching their knee before the thermal image acquisition without pants, socks, shoes, and with light clothing such as a t-shirt on the top. Participants were asked to stand on a designated floor map. The thermograms of the symptomatic knee were captured using a thermographic camera FLIR T1020 (FLIR^®^ Systems, Täby, Sweden), which has 1024 × 768 pixels of resolution and a thermal sensitivity of 0.02 °C. The camera was positioned at 1 m of distance from the subject, adjusted to their patellar height and positioned perpendicular to the knee. An anterior view image was obtained for each patient using the autofocus modality. Then, maintaining the same knee position, an anatomical marker (a 2 cm diameter circular sticker) was placed on the center of the patella and a second anterior view image was obtained to facilitate the precise subsequent localization of the patella in infrared images. The two anterior images (one with and one without the patellar marker) were aligned side by side on the computer screen, and a template indicating the region of interests (ROIs) was centered over the patella of the unmarked image, using the marked image as a guide (Figure 1) [14]. The ROIs were defined as follows: the patellar area was a square 6 cm in width, divided in “medial patella” and “lateral patella” (6 cm high and 3 cm wide each); the “suprapatellar” area was the area 3 cm over the patella; and the “medial” and “lateral” areas were the regions 3 cm under the patella and on its medial and lateral sides, respectively.

The mean temperatures, as well as maximum and minimum temperatures, were extracted using the software ResearchIR (FLIR^®^ Systems, Sweden) for the overall knee area and the 5 ROIs: medial, lateral, medial patella, lateral patella, and suprapatellar.

### 2.2. Statistical Analysis

All continuous data were expressed in terms of the mean and the standard deviation of the mean and range; the categorical data were expressed as frequency and percentages. The Shapiro–Wilk test was performed to test normality of continuous variables. The Levene test was used to assess the homoscedasticity of the data. The repeated measures general linear model (GLM) with Sidak test for multiple comparisons was performed to assess the differences in different areas. The ANOVA test was performed to assess the between groups differences of continuous, normally distributed, and homoscedastic data; the Mann–Whitney non-parametric test was used otherwise. The ANOVA test, followed by the post hoc Sidak test for pairwise comparisons, was performed to assess the among groups differences of continuous, normally distributed, and homoscedastic data; the Kruskal–Wallis non-parametric test, followed by the post hoc Mann–Whitney test with Bonferroni correction for multiple comparisons, was used otherwise. The Spearman rank correlation was used to assess correlations between temperature and continuous data; the Kendall tau rank correlation was used for ordinal data. With 40 patients, a post hoc power equal to 0.9 was obtained with the Kendall’s ordinal correlation between the IKDC objective score and the total mean knee temperature. For all tests, *p* < 0.05 was considered significant. All statistical analyses were performed using SPSS v.19.0 (IBM Corp., Armonk, NY, USA).

## 3. Results

The mean temperatures of the evaluated OA knees are shown in Table 2. The mean temperature of the total knee was 31.9 ± 1.6 °C. Analyzing the mean temperature of the different areas, the patella (both medial and lateral areas) was found to be colder than all other areas of the knee. In particular, the mean temperature of the medial area was higher than medial patella and lateral patella areas (both *p* < 0.0005). The mean temperature of the lateral area was higher than medial patella (*p* = 0.019) and lateral patella areas (*p* = 0.048). The mean temperature of the suprapatellar area was higher than medial patella and lateral patella areas (both *p* < 0.0005). No significant differences were found among the medial, lateral, and suprapatellar areas (*p* = n.s.).

The mean temperature of the total knee negatively correlated with age (rho = −0.380, *p* = 0.016) and positively with BMI (rho = 0.421, *p* = 0.007), with higher temperatures in patients younger and with higher BMI. This correlation was also confirmed for all sub-areas, except for the medial patellar area in relation to age, as shown in Table 3. Males tended to be warmer than females, with higher mean temperatures of the total knee (32.2 ± 1.2 vs. 31.4 ± 0.8, *p* = 0.051), medial area (32.4 ± 1.1 vs. 31.5 ± 0.7 *p* = 0.017), lateral area (32.2 ± 1.1 vs. 31.3 ± 0.9, *p* = 0.019), and suprapatellar area (32.4 ± 1.2 vs. 31.5 ± 0.8 *p* = 0.025), while no significant differences between sexes were found for the patella areas.

**Figure 2:**
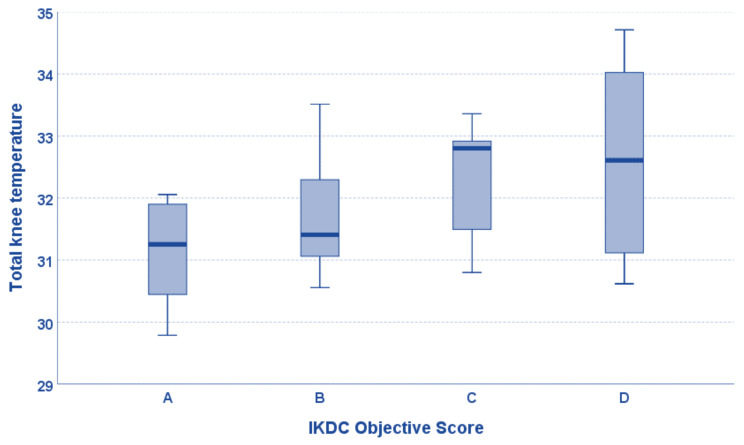
The mean temperature of the total knee positively correlated with the International Knee Documentation Committee (IKDC) objective score (tau = 0.294, *p* = 0.016). Box-and-whisker plots show median values and interquartile ranges.

**Figure 3:**
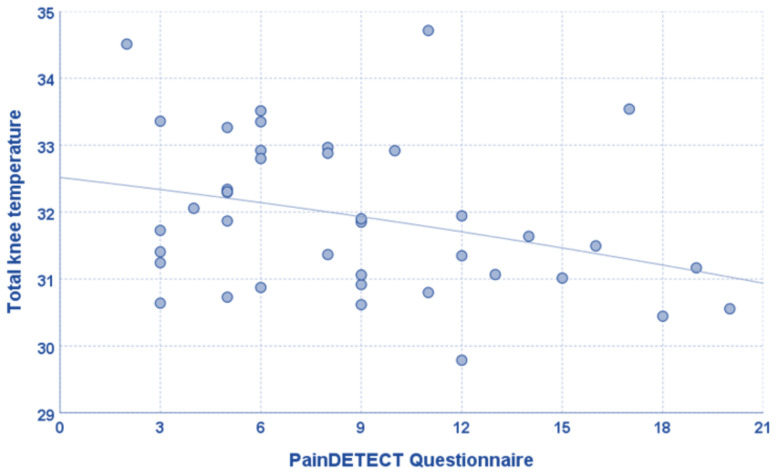
The mean temperature of the total knee positively correlated with the PainDETECT Questionnaire (rho = −0.319, *p* = 0.045).

Regarding clinical scores, the mean temperature of the total knee correlated with the IKDC objective score (tau = 0.294, *p* = 0.016), and this correlation was confirmed for all sub-areas (Figure 2). Patients with neuropathic pain had a lower mean temperature of the medial knee area than patients without neuropathic pain (31.5 ± 1.0 vs. 32.3 ± 1.1, *p* = 0.042), although only a tendency was found for the total knee and the remaining sub-areas. Moreover, the mean temperature of the total knee and the medial area negatively correlated with the PainDETECT Questionnaire (rho = −0.319, *p* = 0.045, rho = −0.366, *p* = 0.020, respectively), while a tendency was found for the other four sub-areas (Figure 3). The mean temperature of the medial knee area correlated with the VAS pain scale (rho = −0.361, *p* = 0.022), while a tendency was found for the mean temperature of the total knee (rho = −0.298, *p* = 0.062), the lateral area (rho = −0.291, *p* = 0.068), and the lateral area (rho = −0.301, *p* = 0.060).

Finally, the mean temperatures of the total knee and other sub-areas were not influenced by other factors such as side, symptom duration, Kellgren–Lawrence grade, previous surgery, and smoke.

## 4. Discussion

The main finding of this study is that the skin temperature of knees affected by OA is influenced by demographic and clinical characteristics of patients, including age, sex, BMI, and objective and subjective scores. In particular, higher joint temperatures were found in younger male patients with higher BMI and worst objective knee scores, while lower temperatures were found in patients affected by neuropathic pain.

The detection of knee temperature through infrared thermography can be useful for better profiling patients with different knee OA patterns. Over the years, the use of infrared thermography has gained a growing interest in the clinical research to evaluate musculoskeletal disorders, quantifying the skin temperature in order to better characterize the properties and course of a specific disease [15,16,17]. The study of joint temperature could potentially improve the diagnosis and therapy of orthopedic pathologies, including knee diseases such as OA [18,19]. A recent systematic review of the literature underlined the correlation between surface skin temperature and joints’ inflammatory and degenerative diseases, including rheumatic pathologies and OA [20]. In particular, a correlation was shown between thermal findings and diseases’ presence and stage, as well as the clinical assessment of disease activity and response to treatment, supporting infrared thermography’s role in the study and management of rheumatic diseases and OA. Nevertheless, evidence on infrared thermography application for knee OA is still limited, especially on elucidating demographic and clinical characteristics that could influence the temperature of the knee in patients affected by symptomatic OA. The current study demonstrated that different factors could influence the skin temperature of symptomatic OA knees.

Age showed a negative correlation with the skin temperature of the knee, with younger patients having higher temperatures compared to older patients. The lower temperature of the knees of elderly patients is ascribable to the decrease in body temperature with aging, probably due to a reduction in basal metabolic rate and a lower muscle component compared to the younger population [21,22,23]. Moreover, a reduction in the overall skin temperature with aging has been justified by the reduction in core temperature in older adults due to a reduction in metabolic processes and to an alteration in the heat dissipation through the skin [22,24,25]. This was reported in healthy subjects by Ferreira et al., who found that young subjects’ limbs’ temperature was higher compared to the elderly subjects’ limbs [26]. The current study confirmed the difference of temperature based on age also for knees affected by symptomatic OA, with lower skin temperatures in older patients considering both the overall knee temperature and most sub-areas. In fact, a further sub-analysis confirmed this correlation for all sub-regions but the region above the patellar bone.

The analysis of knee sub-regions is important when investigating knee OA thermographic patterns. Different temperatures have been detected among the different knee areas, with the area corresponding to the patella reporting the coldest temperature while the medial and suprapatellar areas reporting the highest temperatures. The patella area of the investigated knee OA patients showed a distinct thermal pattern compared to the other areas. In fact, its skin temperature was colder than all other areas of the knee, as previously reported in the literature also in healthy subjects. A description of the thermal image of the normal knee was previously given by several authors who described the thermographic image of a normal knee to be characterized by symmetry in the image of the two knees with an isothermal oval area corresponding to the patella [27,28]. The lower skin temperature of the patellar area could be explained by the fact that the skin tends to be colder above tendons and bones than above muscles [29,30]. Accordingly, the patella represents the coldest area for knee OA patients, and this could be probably linked to the fact that this area of the knee is the furthest from the intra-articular synovium due to the interposition of the patellar bone. In fact, the skin temperature of the knee could reflect the joint inflammatory process, characterized by an increased vascularization of the synovial membrane, which could be easily detected in areas without underlying bone [31].

Other aspects that influenced the skin temperature of the knee OA patients studied were sex and BMI. Neves et al. evaluated the influence of gender and body fat on temperature. They discovered that women exhibit lower values of surface temperature than man on the trunk and upper and lower limbs [32]. The literature data showed that women differ from men in thermal responses to exogenous heat load and heat loss, as well as to endogenous heat load during exercise, because they usually have a larger ratio of body surface to body mass, a greater subcutaneous fat content, and lower exercise capacity. Perhaps also a lower blood volume in women than in men may limit their heat exchange [33]. Moreover, men have a significantly higher muscle mass and lower body fat percentage than women [34]. Finally, this can be also explained by the fact that women have a lower metabolic rate than men [35]. Accordingly, the data emerged from the current study showed that men have higher knee skin temperature than women.

More controversial findings have been found regarding BMI. Higher temperatures were detected for all areas of the knee in patients with higher BMI. This finding is not in line compared to what has been reported in the literature for the overall temperature in obese subjects. In fact, subjects with increased body fat percentage showed lower temperatures of the lower limbs compared to normal-weight individuals [32]. This discrepancy could be explained by the increased inflammatory component that characterized knees of overweight or obese patients who are also affected by OA, probably resulting in an increase of the skin temperature at the OA knee level [36]. In fact, it has been demonstrated that obesity is associated with a chronic inflammatory environment at joint level, which can increase biomarkers of synovial inflammation [37,38,39,40,41]. The obesity-related dyslipidemia can also contribute to OA pathogenesis and increase of inflammation by an increasing matrix metalloproteinases production in joint tissues [42]. Moreover, obese patients present a higher mechanical loading on their joints, resulting in an altered activation of multiple inflammatory pathways, such as interleukin 1-beta (IL-1β) and tumor necrosis factor-alpha (TNF-α) release, chondrocyte apoptosis induction, synovial inflammation, and subchondral bone dysfunction, all contributing to OA [43]. Future studies should investigate the correlation between the skin temperature of the knee and the inflammatory biomarker profile of the affected joint and analyze these findings in relation to the clinical status of the patients.

The clinical evaluation of the knee OA joints in this series showed a significant positive correlation between the skin temperature and the IKDC objective score. This score evaluates objective features of the knee including joint effusion [44]. The current study demonstrated that OA knees with a worse objective clinical status are characterized by a higher temperature, probably related to a high inflammatory component of the joint, with consequent high functional limitations [45]. This finding was similar to a previous study which showed a correlation between higher skin temperature of the knee and worst Western Ontario and McMaster Universities Osteoarthritis (WOMAC) stiffness and function scores [46]. Conversely, the subjective scores analyzed in this case series did not correlate with the skin temperature of the evaluated knee, and the VAS pain score actually showed a negative correlation.

The controversial findings with respect to the pain perception and temperature can be explained by another result of this study, where the pain experience was investigated also in terms of another aspect, the neuropathic pain component. This was investigated with the painDETECT questionnaire, a score evaluating the contribution of neuropathic pain in the pain perceived by the patient [47,48]. A significant contribution of neuropathic pain is present in 23% of patients with knee or hip OA, with a typical symptomatologic pattern with burning pain, shooting pain or lancinating pain, tactile allodynia, and pain patterns [47,49]. Patients with a neuropathic pain showed a lower skin temperature of the knee at medial area compared with patients without neuropathic pain. A possible explanation for this result is that the pain in patients with neuropathic pain is not related to a significant inflammatory component, while it could be due to a central sensitization and an impaired pain modulation [50]. This hypothesis is confirmed by a study conducted by Ohtori S. et al., who found a tendency for negative correlation between the painDETECT score and the amount of joint fluid, with less joint fluid in patients with neuropathic pain [51]. Therefore, the evaluation of patients with symptomatic knee OA but with a “low” temperature should always be investigated for the presence of neuropathic pain, although future targeted studies are needed to better elucidate this aspect.

This study has some limitations. First of all, the sample size could limit the statistical power to better investigate correlations among sub-groups. Therefore, while this is one of the largest studies on symptomatic knee OA patients, future studies with larger populations should confirm the correlation found in the current study. Second, knees were evaluated with an anterior view alone, while further information could be obtained through lateral and posterior thermal acquisitions. Third, a control group of non-symptomatic knee OA patients or non-OA knee patients could have helped understanding the role of thermography in detecting changes of the temperature related to the severity or the presence of OA disease. Another possible weakness is that the evaluation of the neuropathic pain was performed based only on the PainDETECT questionnaire rather than on specific clinical and instrumental exams. Future studies should better investigate the influence of the neuropathic pain component on skin temperature of OA knees. Moreover, the thermographic evaluation of the knee skin temperature could be influenced by the time of the day chosen, and further studies should investigate the behavior of the knee skin temperature during the daytime. Additionally, future studies will have to better characterize the temperature differences of the knee affected by symptomatic knee OA compared to the healthy contralateral one. Finally, the method of thermographic image acquisition and analysis was based on the previous literature, but no method has been described as the gold standard in this field. It is possible that different settings, different lenses, and different devices could be more suitable for such evaluations in the clinical practice. Therefore, future studies should help standardize more the use of thermography for the evaluation of patients with knee OA in order to confirm its potential in identifying different disease patterns both in the research setting and in the clinical practice. This could have the potential to better address patients with knee OA, improving diagnosis, management, and treatment, with a possible socioeconomic and healthcare impact in the future.

## 5. Conclusions

This study demonstrated that the skin temperature of OA symptomatic knees evaluated with infrared thermography is influenced by demographic and clinical characteristics of patients, including age, sex, BMI, and objective and subjective scores. Higher joint temperatures were found in younger male patients with higher BMI and worst objective knee scores, while lower temperatures were found in patients affected by neuropathic pain.

## Figures and Tables

**Table 1:** Included patients’ characteristics.

**Sex (M/W)**	26/14
**Age (years)**	61.3 ± 9.3 [43–75]
**BMI (Kg/m^2^)**	25.2 ± 3.0 [19.9–31.1]
**Side**	Right: 21-Left: 19
**Symptoms duration (months)**	108.5 ± 91.4 [18–372]
**Symptoms onset**	Acute: 13-Chronic: 27
**Previous knee surgery (yes/no)**	22/18
**Smoke (yes/no)**	9/31
**Kellgren–Lawrence grade**	Grade 2: 19 Grade 3: 18 Grade 4: 3
**VAS pain**	5.0 ± 2.4 [1–9]
**IKDC Subjective score**	42.3 ± 15.1 [18.4–81.6]
**IKDC Objective score**	Grade 1: 6 Grade 2: 17 Grade 3: 9 Grade 4: 8
**KOOS Pain**	61.9 ± 19.1 [17–94]
**KOOS Symptoms**	60.5 ± 20.5 [18–100]
**KOOS ADL**	69.9 ± 18.6 [38–100]
**KOOS QoL**	34.5 ± 16.9 [0–75]
**KOOS Sport/Rec**	42.6 ± 17.7 [20–90]
**Tegner score pre-treatment**	2.3 ± 1.2 [1–5]
**PainDETECT Questionnaire**	8.8 ± 4.9 [2–20]
**Neuropathic pain (yes/no)**	8/32

Values are expressed as mean ± standard deviation and [range]. ADL, activities of daily living; BMI, body mass index; IKDC, International Knee Documentation Committee; KOOS, Knee Injury and Osteoarthritis Outcome Score; M, male; QoL, quality of life; Sport/Rec, function in sport and recreation; VAS, visual analogue scale; W, women.

**Table 2:** Temperatures of the evaluated knee areas.

Area	Mean Temperature	Minimum Temperature	Maximum Temperature
**Total knee**	31.9 ± 1.6	30.3 ± 1.1	33.6 ± 1.2
**Medial**	32.1 ± 1.0	30.7 ± 1.0	33.4 ± 1.2
**Lateral**	31.9 ± 1.0	30.5 ± 1.1	33.1 ± 1.1
**Medial Patella**	31.6 ± 1.4	30.9 ± 1.3	32.5 ± 1.4
**Lateral Patella**	31.6 ± 1.5	30.9 ± 1.4	32.6 ± 1.5
**Suprapatellar**	32.1 ± 1.2	30.7 ± 1.1	33.3 ± 1.2

**Table 3:** Temperature correlates with age and BMI.

Area	Age	BMI
**Total knee**	Rho = −0.380, *p* = 0.016	Rho = 0.421, *p* = 0.007
**Medial**	Rho = −0.450, *p* = 0.004	Rho = 0.333, *p* = 0.036
**Lateral**	Rho = −0.387, *p* = 0.014	Rho = 0.365, *p* = 0.020
**Medial Patella**	Rho = −0.265, *p* = 0.098	Rho = 0.461, *p* = 0.003
**Lateral Patella**	Rho = −0.329, *p* = 0.038	Rho = 0.512, *p* = 0.001
**Suprapatellar**	Rho = −0.377, *p* = 0.016	Rho = 0.433, *p* = 0.005

## Data Availability

Not applicable.
